# In the future workplace: how the Dark Triad influences unethical behavior through the Fraud Pentagon among accounting students

**DOI:** 10.3389/fpsyg.2026.1759730

**Published:** 2026-03-25

**Authors:** Fahad Albejaidi, Yasir Hayat Mughal

**Affiliations:** Department of Health Informatics, College of Applied Medical Sciences, Qassim University, Buraydah 51452, Saudi Arabia

**Keywords:** Dark Triad, Fraud Pentagon, Machiavellianism, narcissism, psychopathy, unethical behavior, workplace

## Abstract

**Introduction:**

Corruption is a blight that can infect the economy like termites. The most prevalent sources of corruption in every community are unethical and deceitful behavior. People act unethically for many reasons, the most serious being the Dark Triad. Accounting literature has recently focused on the Dark Triad (narcissism, Machiavellianism, and psychopathy), which can lead to fraudulent and unethical behavior at work. Accounting students’ immoral conduct toward their future professional practices was investigated in this study as a contribution to evolutionary theory and the theory of mind. Professional life involves secrecy, confidentiality, a hidden set of operations, and unethical behavior; this study reveals the hidden links between the infamous Dark Triad traits of narcissism, Machiavellianism, and psychopathy and the misuse of power, resources, and the pervasive web of corruption that ensnares moral behavior. The aim of this study was to investigate the mediating effect of the Fraud Pentagon (motivation, opportunity, capability, rationalization, and intention) on the relationship between Dark Triad traits (narcissism, Machiavellianism, psychopathy) and unethical behavior. Applying a combined view of evolutionary theory and the theory of mind, the study draws on data collected from graduate accounting students in their last semester or year of study.

**Methods:**

A survey research design was used. The study was quantitative in nature, and time-lagged data were collected. We used a three-wave survey design with a 15-day time lag, and data were collected from (*n* = 512) accounting students out of 600. To assess the reliability and validity of the questionnaires, confirmatory factor analysis was conducted in the measurement model developed using PLS-SEM. To test the hypotheses, a structural model was developed.

**Results:**

The results indicate that the Fraud Pentagon significantly mediated the relationship between narcissism, Machiavellianism, and accounting students’ unethical behavior toward their future professional practices.

**Discussion:**

This study established that fraud pentagon has served as a critical cognitive pathway linking dark triad personality traits to unethical behavior of accounting students through lens of evolutionary theory and theory of mind.

## Introduction

A high level of moral character is essential for success in the accounting field, which relies heavily on following the rules and adhering to ethical principles (Aveh et al., 2016). In line with organizational literature, “dark” personality traits and their potential effects on unethical and fraudulent behavior have recently been acknowledged in accounting research (Harrison et al., 2018). The taxonomy is still being worked out, but it appears that callousness is the common denominator among these traits (Jones and Paulhus, 2014). The Dark Triad refers to a cluster of three related personality traits characterized by a deficiency of empathy, manipulation, fraudulence, and immorality. This can lead to unethical and fraudulent acts both in the corporate world and elsewhere (Jones and Paulhus, 2011; Miller, 2016; Serwaa Owusu Mensah et al., 2025). In an era marked by growing ethical scrutiny and regulatory demands, understanding the psychological underpinnings of unethical behavior is not only academically pertinent but also essential for shaping future guardians of financial integrity (Elbæk and Mitkidis, 2023).

While the Dark Triad provides a robust framework for identifying individuals predisposed to unethical conduct, it does not fully explain the cognitive processes through which these personality traits translate into actual unethical decisions (Smith et al., 2021; Antonicelli and Felski, 2024). This study addresses this gap by proposing the Fraud Pentagon as a mediating mechanism that captures how Dark Triad traits shape unethical behavioral intentions through specific cognitive appraisals (Arad and Ghaseminejad, 2025; Liang et al., 2025). In developing economies, such as Pakistan, where corruption is a significant contributor to economic decline (Khan, 2007), it is essential to address the root causes of fraud in corporate environments. To strengthen the contextual grounding, it is pertinent to note that Pakistan ranked 140th out of 180 countries on Transparency International’s Corruption Perceptions Index (2022), and the demand for accounting professionals continues to grow amidst financial sector reforms, highlighting the urgency of ethical preparedness (Mungiu-Pippidi, 2023). Within this context, understanding the psychological pathways to unethical behavior among future accounting professionals carries particular significance (Bailey, 2017, 2019).

This research examines how the Dark Triad personality disposition affects the ethical decision-making of graduate accounting students, considering both current tendencies and potential future impact. It considers how accounting students make decisions through the analysis of Fraud Pentagon beliefs, i.e., motivation, opportunity, rationalization, capability, and intention (Crowe, 2011). As graduate students in their final year are about to enter professional life soon (Bailey, 2017, 2019), addressing these concerns is of utmost significance for the future of the financial industry. This research deliberately focuses on graduate students because they are at a critical juncture and are exposed to an advanced professional curriculum, making them close to entering the workplace; thus, ethical disposition becomes highly relevant for forecasting future unethical professional behavior (Hidajat, 2024; Aulia et al., 2026).

Research into the effect of darker personality characteristics on accounting students’ propensity to engage in unethical behavior in a professional setting is necessary in a number of ways. First, accounting is a highly regulated area where unethical behavior can lead to a whole host of problems not only personally but also for the community at large (Serwaa Owusu Mensah et al., 2025). Understanding what contributes to unethical behavior will assist in fraud prevention and thus protect the public interest. Second, dark personality traits—namely psychopathy, Machiavellianism, and narcissism—are associated with unethical behavior in the workplace (Amos et al., 2022; Harrison and Summers, 2026). Examining how these Dark Triad traits affect accounting students’ unethical professional practices requires further investigation. Third, the “Fraud Pentagon” (motive, opportunity, rationalization, capability, and arrogance) may heighten dishonesty in those accounting students who possess predisposing dark personality traits (Arad and Ghaseminejad, 2025).

Moreover, recent high-profile financial crises in Pakistan have heightened the demand for better ethics and practices in the accounting profession (Rashid et al., 2023), making it especially important to reach out to students in Pakistan who are considering careers in accounting (Anjum, 2021). Additionally, accountants are in high demand in Pakistan; therefore, the selection of candidates who will be trustworthy when they enter the workforce is highly important. To this end, we employed the Fraud Pentagon as a mediating mechanism to examine the relationship between the Dark Triad and unethical behavior. We propose that Dark Triad traits predispose individuals to cognitively appraise situations through the lens of pressure, opportunity, rationalization, capability, and arrogance. This appraisal process, through the activation of the Fraud Pentagon mindset, then directly catalyzes unethical behavioral intentions (Prastiwi et al., 2025). This mediation model integrates dispositional (Dark Triad) and situational-cognitive (Fraud Pentagon) perspectives. To address the unresolved discrepancy in behavioral manifestations, we draw on the theory of biological evolution proposed by Darwin (1964) and the theory of mind (ToM) (Baron-Cohen, 1999).

The theory of biological evolution suggests that living organisms have varying personality traits in terms of behavior, anatomy, and physiology that can be passed down from generation to generation, with varying success rates in survival and replication. Dark personality traits can be understood through this evolutionary lens, as they predict an organism’s ability to adapt and function in society (Furnham et al., 2013) and point to mating tactics as an important driver of their evolution. Specifically, traits like manipulativeness (Machiavellianism) and fearless dominance (psychopathy) may have conferred short-term adaptive advantages in social competition and resource acquisition, potentially explaining their persistence (Amos et al., 2022; Muris et al., 2017). The Fraud Pentagon components, particularly “capability” and “rationalization,” can be viewed as modern cognitive manifestations of these evolved strategies for exploiting social and organizational environments (Prastiwi et al., 2025). This evolutionary perspective explains why Dark Triad traits predispose individuals toward the cognitive patterns captured by the Fraud Pentagon.

However, ToM posits that individuals’ mental states are the foundation for their ability to monitor, anticipate, and regulate their own and others’ behavior and emotions (Gernsbacher and Yergeau, 2019), which can be used as a neutral facilitator for both prosocial and antisocial actions, such as fraud and interpersonal manipulation (Opriș et al., 2025). The relationship between ToM and the Dark Triad is complex and influenced by internal mental states, beliefs, and behavior, potentially enabling the manipulation of ToM skills (Doyle, 2020). From this perspective, individuals high in Dark Triad traits may deploy their ToM capabilities instrumentally to identify vulnerabilities in others, craft plausible rationalizations, and execute manipulative strategies, thereby activating the cognitive pathways specified in the Fraud Pentagon framework. The novelty of the study also lies in the emerging role of the Dark Triad, Fraud Pentagon, and unethical behavior in contributing to evolutionary theory and ToM.

Notably, the major contribution of this investigation is to warn educational institutions and parents about the importance of their attentiveness in developing the character of the young generation at an evolutionary level and toward a mature mindset (Truhan, 2025). For parents, the study may enlighten them about the importance of instilling values such as honesty, integrity, and empathy in their children at an early age. As an added advantage, it can help identify the initial emergence of dysfunctional behavior in youngsters and provide parents with the necessary means to ensure that their children do not fall into the same level of immorality (Ahmad et al., 2025). In a world where institutional trust is becoming increasingly tenuous, the ability to prepare the next generation of accountants not only with the skills necessary to excel in their profession but also with the psychological tools to resist unethical justifications is not only desirable but also absolutely necessary for the integrity of financial systems around the globe (Antonicelli and Felski, 2024). Moreover, the study may also help enhance accounting regulatory frameworks and industry standards that would benefit society and HEIs as well (D’Souza and Lima, 2021).

## Literature review and hypotheses development

### Theoretical perspective

To comprehend the psychological mechanisms leading to unethical behavior, it is important to combine both schools of thought that consider the origins of personality traits and the cognitive mechanisms by which such traits result in action. To elaborate the mediation model, this research builds on two underlying theories, evolutionary theory and theory of mind (ToM), to provide the mediation framework with more extensive explanatory coverage. According to the theory of biological evolution, living organisms possess different personality characteristics in terms of behavior, anatomy, and physiology that may be passed down through generations with varying degrees of success in survival and reproduction (Jonason et al., 2010). This evolutionary perspective can be applied to describe dark personality traits because they predict an organism’s adaptation and functioning in society (Furnham et al., 2013) and point to mating tactics as a key factor in their evolution. In particular, characteristics such as manipulativeness (Machiavellianism) and fearless dominance (psychopathy) may provide short-term adaptive benefits in social competition and resource acquisition, potentially explaining their maintenance (Amos et al., 2022). In this sense, the Dark Triad represents a complex of socially exploitative strategies, whose perhaps maladaptive expressions in the contemporary organizational setting could have once been adaptive strategies for coping with competitive social conditions (Volk et al., 2025). The Fraud Pentagon elements are also considered contemporary cognitive expressions of these evolved strategies, serving as situational triggers and cognitive instruments through which ancient predispositions may manifest in the work environment today (Prastiwi et al., 2025; Dias-Oliveira et al., 2021).

According to ToM, the mental states of people are the basis of their capacity to monitor, predict, and regulate their own and other people’s behavior and feelings (Gernsbacher and Yergeau, 2019). Although ToM skills have commonly been linked to prosocial behavior, they can also be used as an intermediary in both prosocial and antisocial behaviors, including fraud and interpersonal manipulation. Notably, individuals high in the Dark Triad, specifically those exhibiting Machiavellianism and psychopathy, are thought to possess a functional, even superior, ToM that is applied to gain instrumental rather than empathic advantages (Opriș et al., 2025). This cold ToM allows them to model the beliefs, intentions, and vulnerabilities of other people in an effective way, a cognitive ability that is especially effective when it is triggered by perceived opportunities for exploitation (Doyle, 2020). These theoretical approaches provide a consistent basis for the mediation framework. Evolutionary theory is used to understand why dark personality traits are maintained and why these traits predispose people to exploitative strategies, whereas ToM is used to understand the cognitive architecture that enables these exploitative strategies to be implemented with sophistication. The Fraud Pentagon, as the mediating mechanism, represents the situational-cognitive appraisal process that translates these evolutionary predispositions and cognitive capabilities into specific unethical behavioral intentions.

### The Dark Triad, Fraud Pentagon, and unethical behavior

There is a considerable amount of literature that has reported the interconnection between Dark Triad traits and professional misconduct (Little and Handel, 2016). Individuals who receive a high score on these traits tend to be driven by greed, power, and domination concerns and are directed toward unethical means of accomplishing their objectives (Harrison et al., 2018). High levels of Dark Triad traits have also been associated with financial misreporting, embezzlement, and fraudulent schemes (Harrison et al., 2018; Jones and Paulhus, 2014; Jones and Figueredo, 2013).

Narcissism has often been linked to immoral behavior in various settings. The feeling of entitlement makes narcissistic people think that they are entitled to special treatment and that it is their right to break the rules to reach their goals (Alowais and Suliman, 2025). They are obsessed with the desire to be admired, and this can push them to distort their achievements or manipulate facts to sustain their large ego. Studies have associated narcissism with financial misreporting, fraud, and other unethical organizational behavior (Roeser et al., 2016). Narcissistic behavior among accounting students can manifest in the form of distorting information or prioritizing personal success over professional standards. To amplify their egos, narcissists usually justify fraud since they think their extraordinary personality will grant them extraordinary treatment (Liang et al., 2025).

Machiavellianism is arguably the Dark Triad trait that has the most stable and strongest relationship with unethical behavior (Baughman et al., 2012). Machiavellians are sly and subversively manipulative in their quest to achieve material values (Harrison et al., 2018). Their pragmatic belief that the ends justify the means, coupled with their strategic manipulation abilities, makes them highly susceptible to unethical actions when these serve their interests. They do not feel guilty about lies, manipulation, and exploitation of others because it does not cause them as much emotional pain as it would in individuals with stronger empathic instincts. Machiavellianism has been implicated in a number of unethical actions, such as fraud, deceptive negotiation, and readiness to break organizational regulations (Furnham et al., 2013; Smith et al., 2022). Machiavellians take a rational approach to ethical decisions and calculate the benefits against the risks of detection rather than adhering to internal moral standards (Liang et al., 2025).

The main symptoms of psychopathy, such as the absence of empathy, impulsivity, and callousness, establish a direct channel to unethical conduct. Psychopaths find it hard to be concerned about other people, and they can lie or rob others without thinking about the consequences (Furnham et al., 2013). The emotional restraints (guilt, remorse, and concern for others) that prevent unethical action in most people do not bother them (Cima et al., 2010). Their impulsiveness implies that they can follow unethical impulses without considering their repercussions. Studies have attributed psychopathy to various malpractices such as fraud, stealing, and exploitation (Jones and Paulhus, 2011). Psychopaths cannot experience empathy or remorse for their deeds and lack moral restraint, as they possess low moral reasoning skills, which increases their tendency to adhere to personal needs rather than moral values (Cima et al., 2010). Psychopathic personalities among accounting students are likely to increase a readiness to commit academic dishonesty and, consequently, professional misconduct in adulthood (Li et al., 2020). Hence, we propose the following hypotheses:

*H1a*: Narcissism positively predicts unethical behavior.

*H1b*: Narcissism positively predicts the Fraud Pentagon

*H2a*: Machiavellianism positively predicts unethical behavior.

*H2b*: Machiavellianism positively predicts the Fraud Pentagon.

*H3a*: Psychopathy positively predicts unethical behavior.

*H3b*: Psychopathy positively predicts the Fraud Pentagon.

*H3c*: The Fraud Pentagon positively predicts unethical behavior.

### Mediation of the fraud pentagon in the relationship between the Dark Triad and unethical behavior

The concept of motivation (pressure) refers to the reasons that lead individuals to commit fraud, which include monetary pressure, the wish to get status, and the necessity for control (Utami et al., 2019). These motivations are often amplified by Dark Triad traits (Fitri et al., 2019). Similarly, narcissists feel strong pressure to preserve and develop their grandiose self-image, which makes them seek any possible means of gaining acclaim, including immoral ones (Harrison et al., 2018). This constant desire to be admired and validated results in a compulsive drive to achieve success and recognition, which may interfere with ethical concerns when legitimate opportunities are limited (Nurcahyono & Hanum, 2023). Thus, narcissists tend to be more vulnerable to pressure to perform by any means, and hence the motivation aspect of the Fraud Pentagon is especially relevant to them (Chimonaki et al., 2023).

Power, control, and material gain are key motivations that drive Machiavellians and support their pragmatic orientation toward fraud (Utami et al., 2019). Their social interaction strategy implies that they are always aware of opportunities to advance, and the desire to obtain resources and power is one of the central features of their personality structure (Furnham et al., 2013). This aggressive achievement drive, unconstrained by empathic concern, predisposes them to greater susceptibility to fraud-related pressures. Psychopaths are driven by immediate gratification and do not have internal checks that would normally limit their pursuit of selfish goals (Jones and Paulhus, 2011; Hermanson, 2025). Their impulsiveness and sensation-seeking provide them with motivation to attain quick gains without consideration of long-term outcomes. The absence of guilt and regret also ensures that once they are motivated, psychopaths do not have internal restraints that would limit them from pursuing unethical routes to attain gratification (Cima et al., 2010; Liang et al., 2025). From an evolutionary point of view, these motivations are considered adaptive, as they provide individuals with the drive to acquire resources and attain status, which may manifest as fraud motivation in an organizational context (Furnham et al., 2013; Volk et al., 2025). As such, Dark Triad traits contribute to unethical behavior through fraud-related motivation. Hence, we proposed the following hypotheses:

*H4a*: Motivation mediates the relationship between narcissism and unethical behavior.

*H4b*: Motivation mediates the relationship between Machiavellianism and unethical behavior.

*H4c*: Motivation mediates the relationship between psychopathy and unethical behavior.

Opportunity, on the other hand, is defined as the perception that fraud can be executed without detection, which includes the identification of opportunities already present and those that are created (Carré et al., 2020). Dark Triad personality dimensions facilitate both the identification and creation of fraudulent opportunities. Machiavellians’ ability to think several steps ahead allows them to identify opportunities for fraud by recognizing system vulnerabilities and weaknesses in relationships (Idensohn et al., 2025). This ability allows them to perceive opportunities that other people cannot. Research has shown that Machiavellians are skilled at identifying opportunities because they can recognize when systems have weaknesses and how other people can be manipulated (Smith et al., 2022). ToM is also applicable here, as it enables Machiavellians to understand other people’s mental states, which allows them to recognize when others are vulnerable, unlikely to detect misconduct, and susceptible to manipulation (Opriș et al., 2025).

Narcissists’ charisma and persuasive ability provide social opportunities to influence others and access restricted information or resources (Smith et al., 2022). The confidence and self-presentational skills of narcissists may provide access and build trust among others, generating opportunities that would not be accessible to less confident individuals. Narcissists may also be more likely to place themselves in situations where opportunities emerge naturally (Harrison et al., 2018). The fearlessness of psychopaths allows them to access opportunities that would not be accessible to others because they are less sensitive to perceived risks (Carré et al., 2020). While others may be dissuaded from accessing certain opportunities because they may be detected or because they are risky, psychopathic individuals may access such opportunities without hesitation. The impulsiveness of psychopaths may also enable them to identify and access opportunities quickly, without sufficient time to reflect on the ethical implications of the opportunity (Antonicelli and Felski, 2024). Dark Triad personality predispositions may influence individuals to identify, generate, and access fraudulent opportunities, which may increase their unethical intentions. Hence, we propose the following hypotheses:

*H5a*: Opportunity mediates the relationship between narcissism and unethical behavior.

*H5b*: Opportunity mediates the relationship between Machiavellianism and unethical behavior.

*H5c*: Opportunity mediates the relationship between psychopathy and unethical behavior.

Rationalization is the cognitive process by which people justify unethical behavior to themselves in a manner that permits them to retain a positive self-image as moral actors while still engaging in immoral behavior (Garrity, 2023). Dark Triad personality traits may have a major impact on this process. Individuals with Dark Triad traits often lack a solid moral foundation and tend to be more prone to creating self-serving justifications for their behavior (Almagtome and Abo-aljun, 2023). As a result of having a diminished capacity for guilt and empathy, individuals with Dark Triad traits may require fewer rationalizations for their unethical behavior (Alowais and Suliman, 2025).

Machiavellians rationalize their dishonesty because it is in their self-interest, especially if they feel they have been mistreated or are just “playing the game” that everyone else is playing (Almagtome and Abo-aljun, 2023). ToM comes into play here, with “cold” ToM, which is characteristic of individuals high in Machiavellianism, helping them develop rationalizations that are difficult to dispute by effectively predicting how other people would view their justifications (Opriș et al., 2025). Narcissists rationalize their behavior because they believe that they deserve the benefits they are seeking (Smith et al., 2021). This feeling of entitlement provides them an automatic justification for their behavior, as they believe they are above the rules because they are special. They may rationalize their unethical behavior as a way of claiming what they believe they deserve (Antonicelli and Felski, 2024). Psychopaths trivialize the importance of others’ pain and externalize blame (Jones and Paulhus, 2011). They may trivialize the consequences, deny responsibility, and externalize blame to others whom they believe deserve victimization. Their lower empathic concern for others’ pain means that they engage in fewer cognitive activities to rationalize their misconduct. In this manner, the Dark Triad affects ethical conduct by modulating rationalization processes. Hence, we propose the following hypotheses:

*H6a*: Rationalization mediates the relationship between narcissism and unethical behavior.

*H6b*: Rationalization mediates the relationship between Machiavellianism and unethical behavior.

*H6c*: Rationalization mediates the relationship between psychopathy and unethical behavior.

Capability is a personal attribute that includes the personal skills and qualities required to perpetrate fraud, including social skills, knowledge, confidence, and manipulative skills (Padayachee, 2021). Dark Triad traits may positively influence fraud-related capabilities. Machiavellians have impressive capabilities in manipulating others to attain their trust and gain access to the targets (Furnham et al., 2013; Hermanson, 2025). Their strategic capabilities to manipulate others, combined with their ability to remain composed and persuasive, enable them to perpetrate complex frauds that involve managing other people’s perceptions and behaviors. Machiavellians possess the ability to manage impressions and pretend to have feelings and commitment that they do not actually possess (Smith et al., 2022).

The interpersonal skills of narcissists, along with their skills in persuasion, allow them to influence others and achieve their objectives (Gonzalez and Kopp, 2018). The confidence of narcissists can also be very persuasive, allowing them to win the trust of other people. Narcissists may also have the skills needed to present themselves in situations where fraud is being committed. The absence of feelings of regret in psychopaths allows them to effectively execute fraud without feelings of hesitation (Opriș et al., 2025). Unlike other people, psychopaths can execute fraud smoothly without feelings of nervousness, regret, or sympathy, which can interrupt the execution of fraud. The psychopathic personality of these individuals does not allow them to betray fraudulent intentions through visible nervousness (Emerson and Smith, 2026). According to ToM, the capacity to perceive others’ mental states can improve one’s ability to adapt strategies in advance (Pant, 2024). People with the capacity to accurately forecast how others will think and act can adjust their fraudulent strategies to match these forecasts, thus increasing their success rate. From an evolutionary viewpoint, these capacities are specialized social abilities that, while being useful in a competitive ancestral environment, now emerge as fraud capabilities in an organizational context (Volk et al., 2025). The Dark Triad traits, therefore, impact unethical behavior through their effect on fraud capability. Hence, we propose the following hypotheses:

*H7a*: Capability mediates the relationship between narcissism and unethical behavior.

*H7b*: Capability mediates the relationship between Machiavellianism and unethical behavior.

*H7c*: Capability mediates the relationship between psychopathy and unethical behavior.

Fraudulent intention is the premeditated desire to commit fraud or the tendency to commit it when the opportunity arises (Jones and Paulhus, 2014). Intention is the interface between cognition and action, and it is the juncture at which an individual makes the decision to engage in unethical conduct. While the Dark Triad directly affects the formation of fraudulent intentions (Srirejeki et al., 2023), the egocentric dispositions, absence of empathy, and tendency to exploit others in individuals with high Dark Triad traits result in stronger fraudulent intentions than in the general population (Cima et al., 2010). The absence of empathy and the tendency to be indifferent to the welfare of others eliminate a crucial inhibitor in the formation of intentions.

Individuals with psychopathy lack the capacity to empathize and feel remorse and therefore develop intentions to commit fraud without the inhibitory effect of moral concerns. Studies have shown that individuals with psychopathy lack moral reasoning and therefore exhibit utilitarian responses to moral dilemmas (Cima et al., 2010; Latif et al., 2026). The intentions developed by such individuals are influenced by the anticipated benefits to the individual rather than by moral obligations. Machiavellians consciously form intentions to manipulate others when it is in their best interest to do so. Machiavellians’ intention formation is a calculated process in which they consider the potential benefits and risks involved (Murthy and Gopalkrishnan, 2023). Unlike the impulsive intention formation of psychopaths, Machiavellians’ intentions are the product of a strategic analysis of the situation. In fact, narcissists form intentions to attain acclaim using any means necessary. If they fail to attain the recognition they need using honest means, they form intentions to attain recognition by deceit or exploitation (Harrison et al., 2018; Latif et al., 2026). Their sense of entitlement is the driving force behind their intention formation. From a biological point of view, the intentions formed by these individuals are a product of a strategic approach to resource acquisition through social exploitation (Smith et al., 2022). Therefore, Dark Triad personality traits lead to unethical behavior through the development of fraudulent intentions. Hence, we propose the following hypotheses:

*H8a*: Intention mediates the relationship between narcissism and unethical behavior.

*H8b*: Intention mediates the relationship between Machiavellianism and unethical behavior.

*H8c*: Intention mediates the relationship between psychopathy and unethical behavior.

*H9a*: The Fraud Pentagon mediates the relationship between narcissism and unethical behavior

*H9b*: The Fraud Pentagon mediates the relationship between Machiavellianism and unethical behavior.

*H9c*: The Fraud Pentagon mediates the relationship between psychopathy and unethical behavior.

## Materials and methods

### Participants

The demographic information of students includes sex, with 392 (76.56%) male students and 120 (23.43%) female students. We divided age into three categories: 127 (24.80%) respondents were between 18 and 20 years of age, 235 (45.89%) were between 21 and 23 years of age; and 150 (29.29%) were aged 24 years of age or older. With regard to education level, 48 (11.32%) students were undergraduate students, 390 (76.17%) were master’s students, and 74 (14.45%) were MPhil students.

### Procedure

Non-probability convenience sampling was used. The advantage of convenience sampling is that any respondent who is available and willing to participate in the survey and provide data can be included (Sekaran and Bougie, 2016). The survey was administered to a total of 600 accounting students from both public and private sector HEIs in southern Pakistan. Only undergraduate students in their last two semesters and postgraduate students in master’s and Master of Philosophy (MPhil) programs in their last two semesters were selected as participants. We invited 600 participants to complete this survey. We distributed questionnaires among 600 students and received a total of 512 responses after eliminating incomplete questionnaires. The students were contacted to obtain their consent to complete the questionnaire. We sent a communication to students assuring them of complete confidentiality (Podsakoff et al., 2012). The questionnaire was provided to those who volunteered to take part in the study. The questionnaires were distributed via university emails and WhatsApp messages. The sampling selection was random, and a three-wave time-lagged strategy was used to avoid common method bias (CMB). The time lag between waves was 15 days. In the first wave, students provided demographic details and responded to questions on the Dark Triad (narcissism, Machiavellianism, and psychopathy). We received 530 responses in the first wave. In the second wave, we sent reminders through email and at the end of class to increase the response rate. In the third wave, we received responses to questions on mediating variable Fraud Pentagon, and we received 527 responses. Later in the third wave, we received responses to questions about the dependent variable (unethical behavior). We received 522 responses, after eliminating responses with missing answers on some questions and incomplete student ID details.

### Instruments

*The Dark Triad (first wave):* The Dark Triad was measured using the Short Dark Triad (SD3) scale developed by Jones and Paulhus (2014), which includes three subclinical dimensions—narcissism, Machiavellianism, and psychopathy—and contains a total of 27 items. The constructs were measured on a seven-point Likert scale ranging from 1 (*strongly disagree*) to 7 (*strongly agree*). The alpha coefficient for the scale was 0.914 for narcissism, 0.913 for Machiavellianism, and 0.903 for psychopathy.

*Fraud Pentagon (second wave):* The Fraud Pentagon consists of five dimensions—opportunity, motivation, rationalization, capability, and intentions—adopted from Harrison et al. (2018) and comprising a total of 12 items. All items were measured on a seven-point Likert scale ranging from 1 (*strongly disagree*) to 7 (*strongly agree*). The alpha values for the scales were 0.831 for opportunity, 0.823 for motivation, 0.815 for rationalization, 0.831 for capability, and 0.818 for intentions. In this study, participants were given scenario examples related to Fraud Pentagon intentions for data collection. They were instructed to imagine themselves promoting a mobile phone and corresponding with potential customers. They were told that exaggerating the phone’s condition could increase their profit from the sale.

*Unethical Behavior (Third wave):* Accounting students’ unethical behavior was measured using an eight-item scale adopted from Bailey (2017). The items were measured on a seven-point Likert scale ranging from 1 (*strongly disagree*) to 7 (*strongly agree*). The alpha coefficient for the instrument was 0.924. Unethical behavior was also measured using scenario-based examples. Participants were asked to respond to the items by assuming that they are working in business management and that the company’s performance was below expectations, prompting them to consider improving the company’s financial statements.

### Data analysis

**Figure 1:**
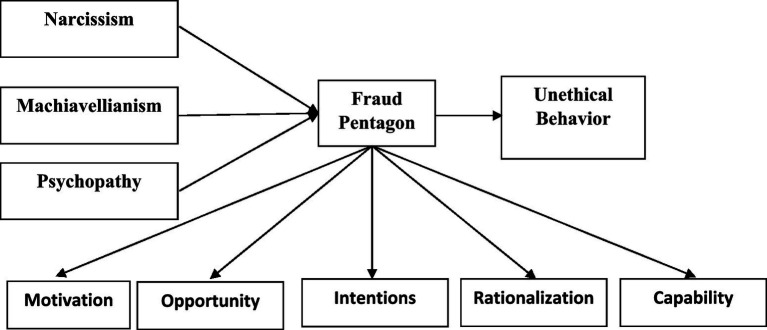
Research model.

**Figure 2:**
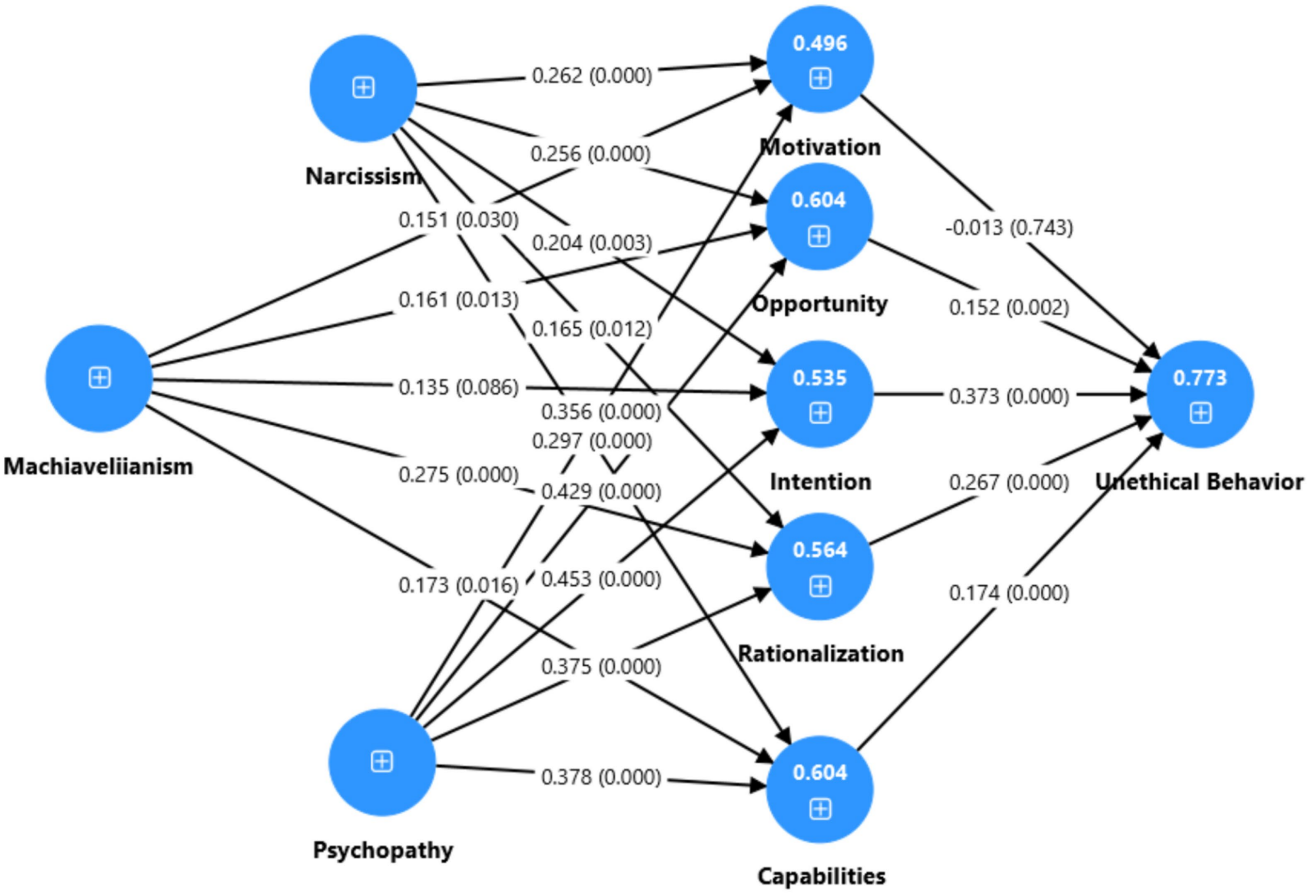
Structural model.

Based on the literature review and theoretical associations, a research model was designed, as summarized in Figure 1. This study adopted a quantitative survey research design (Sekaran and Bougie, 2016). A cross-sectional data collection method was used, while different statistical tests were applied for hypothesis testing. PLS-SEM is commonly used for non-normal data. All the structural and measurement models were developed using PLS-SEM. In this study, we used complex mediation modeling, which was not achievable using SPSS alone, as it was not possible to test the entire study model simultaneously. Therefore, structural equation modeling (SEM) was used. To address this issue and test the model, two alternative options were available. The first was covariance-based SEM (CB-SEM) software, i.e., Mplus, LISREL, and AMOS, or the use of WarpPLS and PLS-SEM. PLS-SEM has many advantages for users, i.e., data with relatively small sample sizes can be analyzed, and formative and complex models, such as mediation models, can also be examined using PLS-SEM. For assessing mediation models, PLS-SEM is considered a reliable analytical technique, as it places few assumptions on sample size, data normality, and independence. Scale validity and reliability were also examined using factor loadings, average variance extracted (AVE), composite reliability (CR), and Cronbach’s alpha values (Hair et al., 2017; Ringle et al., 2020). Moreover, Ramayah et al. (2018) argue that many researchers analyze discriminant validity by using the criterion proposed by Henseler et al. (2015). Convergent and discriminant validity were assessed in the measurement model. Convergent validity was used to assess whether measurement items reflected the same construct, while discriminant validity was used to examine whether constructs were distinct from each other (Ramayah et al., 2018). Discriminant validity was reported using the heterotrait–monotrait (HTMT) ratio. According to Black and Babin (2019), the cut-off value for the HTMT ratio is less than 1. The researchers then tested the study hypotheses using the structural model (Ramayah et al., 2018) (see Figure 2).

## Results

### Preliminary analysis

To analyze the data, we used the PLS-SEM approach (Ringle et al., 2020). Table 1 reports the means, standard deviations, and relationships of all study variables using SPSS. Furthermore, Table 1 shows the correlations among the variables and their respective constructs. From the analysis of the results, it is revealed that for the Fraud Pentagon and its facets, the highest mean value is recorded for motivation (*M* = 5.712), followed by opportunity (*M* = 5.710) and capability (*M* = 5.654), whereas the lowest score is recorded for intentions (*M* = 5.522). The Fraud Pentagon as a composite variable has a mean value of *M* = 5.635. Among the Dark Triad and its facets, the highest mean score is recorded for narcissism (*M* = 5.516) and the lowest for psychopathy (*M* = 5.414). Among the demographic variables, the highest mean score is recorded for age (*M* = 2.040) and the lowest for sex (*M* = 1.240). Overall, the analysis of mean values indicates that the highest score among the continuous variables is recorded for motivation and the lowest for psychopathy. Narcissism is not significantly related to any of the variables, Machiavellianism and psychopathy are moderately related to all variables at the *p* < 0.01 level. Unethical behavior, intentions, rationalization, capability, opportunity, and motivation are highly correlated at the *p* < 0.01 level (see Table 1).

**Table 1:** Mean, standard deviation, and intercorrelations of the study variables.

Variables	*Mean*	*SD*	1	2	3	4	5	6	7	8	9	10	11	12	13
1-Age	2.040	0.735	1												
2-Sex	1.240	0.431	−0.128**	1											
3-Qualification	2.050	0.486	0.059	−0.058	1										
4-Narcissism	5.516	0.946	−0.010	0.083	0.012										
5-Machiavellianism	5.450	0.956	0.018	0.074	0.009	0.061									
6-Psychopathy	5.414	0.857	−0.003	0.091^*^	0.012	0.005	0.707^**^								
7-Unethical behavior	5.464	0.988	−0.033	0.127^**^	−0.001	0.042	0.703^**^	0.732^**^							
8-Intentions	5.522	1.007	−0.048	0.098^*^	−0.054	0.047	0.632^**^	0.647^**^	0.700^**^						
9-Rationalization	5.577	1.019	−0.035	0.042	−0.007	0.039	0.650^**^	0.695^**^	0.703^**^	0.807^**^					
10-Capability	5.654	1.015	−0.028	0.052	−0.002	0.015	0.705^**^	0.705^**^	0.719^**^	0.801^**^	0.778^**^				
11-Opportunity	5.710	1.002	−0.031	0.071	0.003	0.010	0.686^**^	0.697^**^	0.734^**^	0.795^**^	0.775^**^	0.814^**^			
12-Motivation	5.712	0.974	0.008	0.080	0.033	0.020	0.633^**^	0.635^**^	0.658^**^	0.722^**^	0.733^**^	0.730^**^	0.816^**^		
13-Fraud Pentagon	5.635	0.915	−0.030	0.075	−0.006	0.029	0.725^**^	0.741^**^	0.771^**^	0.866^**^	0.889^**^	0.904^**^	0.933^**^	0.935^**^	0.899^**^

Indicators, sex, age, qualification, narcissism, Machiavellianism, psychopathy, Fraud Pentagon (intentions, rationalization, capability, opportunity, motivation), unethical behavior, and Dark Triad.

**Correlation is significant at the 0.01 level (two-tailed).

*Correlation is significant at the 0.05 level (two-tailed).

Table 2 explains the measurement model results for narcissism (NRC), Machiavellianism (MAC), psychopathy (PSY), Fraud Pentagon (FP), and unethical behavior (UB). Field (2013) and Taber (2018) suggest that a high alpha indicates relatively high internal consistency of measuring instruments. Hair et al. (2017) stated that the threshold for factor loadings must be >0.70, composite reliability (CR) and Cronbach’s alpha must be higher than 0.70, average variance extracted (AVE) must be >0.50, and VIF must be less than 5. Table 2 presents the findings of confirmatory factor analysis (CFA). It is evident that Cronbach’s alpha, CR, AVE, and VIF meet the required thresholds. In addition, the factor loadings of all items for their respective constructs meet the threshold of 0.70, except narcissism items 1 and 2, which are less than 0.70; however, the overall construct reliability and convergent validity meet the cut-off level, and therefore, there was no need to exclude these items. Based on the results discussed above, the measurement instrument used in this study is reliable and valid. Discriminant validity was examined using the *HTMT* ratio and the Fornell–Larcker criterion (Fornell and Larcker, 1981) to assess whether constructs are distinct from each other (Henseler et al., 2015; Ramayah et al., 2018). Henseler et al. (2015) suggested that HTMT values should be less than 0.835, whereas Hair et al. (2017) suggested that the value should be less than 1. Table 3 presents the *HTMT* and Fornell–Larcker values, which meet the recommended thresholds except for one value. Hence, the instrument is considered reliable and valid. Narcissism, Machiavellianism, psychopathy, and the Fraud Pentagon (five dimensions) explain the variance in unethical behavior (*R*^2^ = 0.773), i.e., 77.3% of the variance.

**Table 2:** Measurement model.

Variables	Loadings	CR(rho_a)	CR (rho_c)	AVE	*α*	VIF
Psychopathy						
Psychopathy-1	0.727	0.904	0.921	0.565	0.903	1.861
Psychopathy-2	0.743					1.907
Psychopathy-3	0.718					1.756
Psychopathy-4	0.752					1.926
Psychopathy-5	0.795					2.213
Psychopathy-6	0.730					1.821
Psychopathy-7	0.781					2.126
Psychopathy-8	0.768					1.952
Psychopathy-9	0.744					1.884
Machiavellianism						1.861
Machiavellianism-1	0.793	0.914	0.930	0.623	*0.913*	2.129
Machiavellianism-2	0.777					2.051
Machiavellianism-3	0.813					2.291
Machiavellianism-4	0.777					2.034
Machiavellianism-5	0.804					2.202
Machiavellianism-6	0.812					2.241
Machiavellianism-7	0.772					1.994
Machiavellianism-8	0.765					1.902
Narcissism						2.129
Narcissism-1	0.635	0.919	0.929	0.595	*0.914*	1.469
Narcissism-2	0.682					1.701
Narcissism-3	0.760					2.004
Narcissism-4	0.802					2.319
Narcissism-5	0.806					2.279
Narcissism-6	0.791					2.15
Narcissism-7	0.834					2.529
Narcissism-8	0.819					2.406
Narcissism-9	0.791					2.154
Unethical behavior						
Unethical-1	0.778	0.925	0.938	0.654	0.924	2.066
Unethical-2	0.812					2.335
Unethical-3	0.799					2.223
Unethical-4	0.799					2.200
Unethical-5	0.809					2.436
Unethical-6	0.863					2.945
Unethical-7	0.808					2.279
Unethical-8	0.798					2.225
Fraud Pentagon						
Motivation						
Motivation-1	0.858	0.823	0.895	0.739	0.823	1.857
Motivation-2	0.863					1.866
Motivation-3	0.857					1.834
Opportunity						
Opportunity-1	0.859	0.832	0.899	0.748	0.831	1.880
Opportunity-2	0.880					2.042
Opportunity-3	0.855					1.843
Rationalization	0.845					
Rationalization-1	0.843	0.816	0.890	0.730	0.815	1.741
Rationalization-2	0.866					1.865
Rationalization-3	0.853					1.800
Capability						
Capability-1	0.870	0.832	0.899	0.748	0.831	1.930
Capability-2	0.863					1.929
Capability-3	0.861					1.879
Intentions						
Intentions-1	0.867	0.818	0.892	0.733	0.818	1.886
Intentions-2	0.848					1.759
Intentions-3	0.853					1.816

**Table 3:** Discriminant validity.

HTMT ratios	1	2	3	4	5	6	7	8	9
Intention									
Capability	0.942								
Machiavellianism	0.749	0.806							
Motivation	0.893	0.985	0.732						
Narcissism	0.730	0.805	0.878	0.729					
Opportunity	0.940	1.019	0.799	0.999	0.786				
Psychopathy	0.816	0.831	0.849	0.762	0.778	0.847			
Rationalization	0.937	0.981	0.806	0.891	0.753	0.989	0.819		
Unethical behavior	0.942	0.909	0.797	0.828	0.764	0.906	0.868	0.929	

Fornell–Larcker	1	2	3	4	5	6	7	8	9
Intention	0.856								
Capability	0.777	0.865							
Machiavellianism	0.648	0.703	0.789						
Motivation	0.732	0.815	0.636	0.860					
Narcissism	0.634	0.704	0.805	0.636	0.772				
Opportunity	0.775	0.846	0.698	0.827	0.690	0.865			
Psychopathy	0.702	0.722	0.772	0.658	0.710	0.735	0.751		
Rationalization	0.764	0.809	0.697	0.731	0.653	0.814	0.704	0.854	
Unethical behavior	0.821	0.798	0.734	0.723	0.704	0.796	0.793	0.808	0.809

### Hypothesis testing

#### Direct path results

Table 4 presents direct effects of predictors on criterion variables. It is evident from Table 4 that narcissism significantly predicts unethical behavior (UEB) (*β* = 0.188, *SE* = 0.060, *t* = 3.123, *LLCI* = 0.075, *ULCI* = 0.312, *p* < 0.05). In addition, narcissism has a significant effect on the Fraud Pentagon (FP) (*β* = 0.260, *SE* = 0.061*, t* = 4.249, *LLCI* = 0.146, *ULCI* = 0.388, *p* < 0.01). This implies that a one-unit increase in narcissism is associated with a 0.260 increase in FP and a 0.188 increase in UEB. Hence, H1a and H1b are supported. Similarly, Machiavellianism also significantly and positively predicts UEB and FP (*β* = 0.176, *SE* = 0.071, *t* = 2.483, *LLCI* = 0.038, *ULCI* = 0.317, *p* < 0.05; *β* = 0.196, *SE* = 0.067, *t* = 2.929, *LLCI* = 0073, *ULCI* = 0.332, *p* < 0.05). This indicates that a one-unit increase in Machiavellianism is associated with a 0.176 increase in UEB and a 0.916 increase in FP. Thus, H2a and H2b are supported. Similarly, psychopathy significantly predicts UEB and FP (*β* = 0.528, *SE* = 0.063, *t* = 8.424, *LLCI* = 0.407, *ULCI* = 0.655, *p* < 0.01; *β* = 0.437, *SE* = 0.057, *t* = 7.620, *LLCI* = 0.327, *ULCI* = 0.553, p < 0.01). A one-unit increase in psychopathy is associated with increases of 0.528 in UEB and 0.437 in FP. Therefore, H3a and H3b are supported. The most dominant impact is recorded for FP on UEB (*β* = 0.866, *SE* = 0.016, *t* = 52.612, *LLCI* = 0.828, *ULCI* = 0.894, *p* < 0.01). This indicates that a one-unit increase in FP is associated with an increase of 0.866 in UEB. Hence, H3c is accepted.

**Table 4:** Direct effects.

Relationships	*β*	*SE*	*T*	*p*	LLCI	ULCI	Support
(H1a) Narcissism ➔ Unethical Behavior	0.188	0.060	3.123	0.002	0.075	0.312	Yes
(H1b) Narcissism ➔ Fraud Pentagon	0.260	0.061	4.249	0.000	0.146	0.388	Yes
(H2a) Machiavellianism ➔ Unethical Behavior	0.176	0.071	2.483	0.013	0.038	0.317	Yes
(H2b) Machiavellianism ➔ Fraud Pentagon	0.196	0.067	2.929	0.003	0.073	0.332	Yes
(H3a) Psychopathy ➔ Unethical Behavior	0.528	0.063	8.424	0.000	0.407	0.655	Yes
(H3b) Psychopathy ➔ Fraud Pentagon	0.437	0.057	7.620	0.000	0.327	0.553	Yes
(H3c) Fraud Pentagon ➔ Unethical Behavior	0.866	0.016	52.612	0.000	0.828	0.894	Yes

#### Mediation results

Table 5 presents findings of the indirect effects of attributes of the Fraud Pentagon on the Dark Triad (facets) and unethical behavior. Motivation is not found to be a significant mediator between narcissism, Machiavellianism, psychopathy, and unethical behavior (*p* > 0.05). Thus, H4a, H4b, and H4c are not supported. Further analysis of the results revealed that opportunity mediates the relationship between narcissism and UEB, (*β* = 0.039, SE = 0.017, *t* = 2.285, LLCI = 0.012, ULCI = 0.080, *p* < 0.05); thus, H5a is supported. On the other hand, opportunity does not mediate the relationship between Machiavellianism and unethical behavior (*β* = 0.024, SE = 0.013, *t* = 1.868, LLCI = 0.006, ULCI = 0.059, *p* > 0.05); thus, H5b is not supported. Opportunity significantly mediates the relationship between psychopathy and UEB (*β* = 0.065, SE = 0.024, *t* = 2.759, LLCI = 0.022, ULCI = 0.116, *p* < 0.05); thus, H5c is supported. For rationalization, the results show significant mediation between narcissism, Machiavellianism, psychopathy, and UEB (*β* = 0.044, SE = 0.019, *t* = 2.365, LLCI = 0.013, ULCI = 0.086, *p* < 0.05; *β* = 0.074, SE = 0.022, *t* = 3.289, LLCI = 0.036, ULCI = 0.126, *p* < 0.01; *β* = 0.100, SE = 0.025, *t* = 3.974, LLCI = 0.059, ULCI = 0.158, *p* < 0.01, respectively). Hence, H6a, H6b, and H6c are supported. Similarly, capability also mediates the relationship between narcissism, Machiavellianism, psychopathy, and UEB (*β* = 0.052, SE = 0.018, *t* = 2.860, LLCI = 0.021, ULCI = 0.094, *p* < 0.01; *β* = 0.030, SE = 0.015, *t* = 1.991, LLCI = 0.007, ULCI = 0.068, *p* < 0.01; *β* = 0.066, SE = 0.021, *t* = 3.148, LLCI = 0.030, ULCI = 0.113, *p* < 0.01). Hence, H7a, H7b, and H7c are supported. Similarly, intention acts as mediator between narcissism and UEB (*β* = 0.076, SE = 0.027, *t* = 2.856, LLCI = 0.028, ULCI = 0.131, *p* < 0.01); thus, H8a is supported. On the contrary, intention does not significantly mediate the relationship between Machiavellianism and UEB (*β* = 0.050, SE = 0.030, *t* = 1.658, LLCI = −0.005, ULCI = 0.113, *p* > 0.05); hence, H8b is not supported. Further analysis of the results revealed that intention mediates the relationship between psychopathy and UEB (*β* = 0.169, SE = 0.032, *t* = 5.356, LLCI = 0.113, ULCI = 0.240, *p* < 0.01). Hence, H8c is supported. The Fraud Pentagon significantly mediates the relationship between narcissism and unethical behavior (*β* = 0.225, *SE* = 0.053, *t* = 4.223, *LLCI* = 0.126, *ULCI* = 0.338, *p* < 0.01). In addition, FP also mediates the relationships between Machiavellianism and unethical behavior (*β* = 0.170, *SE* = 0.058, *t* = 2.931, *LLCI* = 0.063, *ULCI* = 0.286, *p* < 0.05) and psychopathy and unethical behavior (*β* = 0.379, *SE* = 0.051, *t* = 7.354, *LLCI* = 0.281, *ULCI* = 0.482, *p* < 0.01). Hence, H9a, H9b, and H9c are supported.

**Table 5:** Indirect effects.

Relationships	*β*	*SE*	*T*	*p*	LLCI	ULCI	Support
(H4a) Narcissism ➔ Motivation➔ UEB	−0.003	0.011	0.312	0.755	−0.028	0.017	No
(H4b) Machiavellianism ➔ Motivation➔ UEB	−0.002	0.007	0.300	0.764	−0.019	0.009	No
(H4c) Psychopathy ➔ Motivation➔ UEB	−0.005	0.015	0.323	0.746	−0.036	0.022	No
(H5a) Narcissism ➔ Opportunity ➔ UEB	0.039	0.017	2.285	0.022	0.012	0.080	Yes
(H5b) Machiavellianism➔ Opportunity ➔ UEB	0.024	0.013	1.868	0.062	0.006	0.059	No
(H5c) Psychopathy➔ Opportunity➔ UEB	0.065	0.024	2.759	0.006	0.022	0.116	Yes
(H6a) Narcissism ➔ Rationalization ➔ UEB	0.044	0.019	2.365	0.018	0.013	0.086	Yes
(H6b) Machiavellianism➔ Rationalization ➔ UEB	0.074	0.022	3.289	0.001	0.036	0.126	Yes
(H6c) Psychopathy➔ Rationalization ➔ UEB	0.100	0.025	3.974	0.000	0.059	0.158	Yes
(H7a) Narcissism➔Capability➔ UEB	0.052	0.018	2.860	0.004	0.021	0.094	Yes
(H7b) Machiavellianism ➔ Capability➔ UEB	0.030	0.015	1.991	0.047	0.007	0.068	Yes
(H7c) Psychopathy ➔ Capability➔ UEB	0.066	0.021	3.148	0.002	0.030	0.113	Yes
(H8a) Narcissism➔ Intention ➔ UEB	0.076	0.027	2.856	0.004	0.028	0.131	Yes
(H8b) Machiavellianism➔ Intention➔ UEB	0.050	0.030	1.658	0.097	−0.005	0.113	No
(H8c) Psychopathy➔ Intention➔ UEB	0.169	0.032	5.356	0.000	0.113	0.240	Yes
(H9a) Narcissism ➔ Fraud Pentagon ➔ UEB	0.225	0.053	4.223	0.000	0.126	0.338	Yes
(H9b) Machiavellianism ➔ Fraud Pentagon ➔ UEB	0.170	0.058	2.931	0.003	0.063	0.286	Yes
(H9c) Psychopathy ➔ Fraud Pentagon ➔ UEB	0.379	0.051	7.354	0.000	0.281	0.482	Yes

#### Assessment of endogeneity

Endogeneity is one of the greatest threats to management researchers’ ability to correctly specify models and make causal claims (Certo et al., 2018; Wolfolds and Siegel, 2019). We, therefore, assessed potential endogeneity following Hult et al. (2018). Endogeneity occurs when a model is misspecified, a path is omitted, or when there is systematic error in the measurement of focal variables (e.g., Bergh et al., 2019; Bliese and Ployhart, 2002). Table 4 shows that none of the Gaussian copulas (i.e., narcissism, Machiavellianism, psychopathy, and Fraud Pentagon) showed endogeneity. In the case of the dependent variable unethical behavior, the results also yielded no significant copulas. Using Smart PLS 4, we checked all other combinations of Gaussian copulas included in the model, and none were significant (Table 6). We conclude that endogeneity is not present in this study, indicating the robustness of the structural model.

**Table 6:** Endogeneity test.

Constructs	Coefficient	*p*-value
One copula		
GC (Narcissism)*	0.033	0.247
GC (Machiavellianism)*	0.013	0.403
GC(Psychopathy)*	0.015	0.374
GC (Fraud Pentagon) *	−0.060	0.109
Two copula		
Narcissism		
GC (Narcissism)*	0.036	0.275
GC (Machiavellianism)*	−0.005	0.466
GC (Narcissism)*	0.032	0.305
GC (Psychopathy)*	0.004	0.479
GC (Narcissism)*	0.076	0.122
GC (Fraud Pentagon) *	−0.093	0.075
Machiavellianism		
GC (Machiavellianism)*	−0.005	0.466
GC (Narcissism)*	0.036	0.275
GC (Machiavellianism)*	0.004	0.475
GC (Psychopathy)*	0.017	0.413
GC (Machiavellianism)*	0.050	0.229
GC (Fraud Pentagon) *	−0.079	0.099
Psychopathy		
GC (Psychopathy)*	0.004	0.479
GC (Narcissism)*	0.032	0.305
GC (Psychopathy)*	0.017	0.413
GC (Machiavellianism)*	0.004	0.475
GC (Psychopathy)*	0.074	0.197
GC (Fraud Pentagon) *	−0.092	0.100
Fraud Pentagon		
GC (Fraud Pentagon) *	−0.093	0.075
GC (Narcissism)*	0.076	0.122
GC (Fraud Pentagon) *	−0.079	0.099
GC (Machiavellianism)*	0.05	0.229
GC (Fraud Pentagon) *	−0.092	0.100
GC (Psychopathy)*	0.074	0.197
Three copula		
GC (Narcissism)*	0.034	0.300
GC (Machiavellianism)*	−0.008	0.456
GC (Psychopathy)*	0.007	0.466
GC (Machiavellianism)*	0.028	0.348
GC (Psychopathy)*	0.062	0.254
GC (Fraud Pentagon) *	−0.098	0.094
Four copula		
GC (Narcissism)*	0.065	0.176
GC (Machiavellianism)*	0.016	0.416
GC (Psychopathy)*	0.047	0.312
GC (Fraud Pentagon) *	−0.123	0.057

Assessment of endogeneity test using the Gaussian copula approach (dependent variable: unethical behavior).

## Discussion

This research makes a significant contribution to the accounting ethics and behavioral forensics literature, as it empirically investigates the mediating effect of the Fraud Pentagon in the relationship between Dark Triad personality traits and unethical behavioral intentions among future professionals. Although previous studies have identified the connections between Dark Traits and unethical behavior and have also confirmed the Fraud Pentagon as a diagnostic framework (Harrison et al., 2018; Bailey, 2019; Crowe, 2011), this study proposes a unified psychological model in which the disposition inclinations are translated into unethical intentions through a specific, situation-activated cognitive state. This process is further explained through the theoretical perspectives of evolutionary theory and ToM, providing a more mechanism-based explanation of why individuals with the dark personality tendencies are predisposed to ethical failure (Prastiwi et al., 2025; Arad and Ghaseminejad, 2025; Lyons et al., 2019).

Our findings strongly confirm this integrative model in that the Fraud Pentagon is a significant cognitive process through which all three Dark Triad traits are positively related to unethical behavioral intentions in accounting students, and the findings also show that narcissism, Machiavellianism, and psychopathy all predict the use of fraudulent cognitive processes as well as behavioral intentions to engage in unethical behavior (Smith et al., 2021; Antonicelli and Felski, 2024; Liang et al., 2025; Poless et al., 2018). The Fraud Pentagon, in turn, becomes a strong proximal process, and these results complement previous studies by revealing that the interdependence between personality and unethical behavior works significantly through the cognitive evaluation embodied by this model (Reskino et al., 2021; Dias-Oliveira et al., 2021).

In the case of narcissism, the results indicate that fraudulent motives can be described by a variety of cognitive processes occurring within the Fraud Pentagon, where the sense of entitlement and grandiose self-image help narcissistic individuals transfer their desire to act unethically into this framework through the production of self-serving rationalizations that shield their inflated self-concept, overestimate their ability to succeed in committing fraud, and develop strong intentions to do so (Alowais and Suliman, 2025; Shengbo et al., 2022; Tang and Tang, 2010; Wang et al., 2022). However, motivation was not shown to be a significant driver of narcissism; thus, the urge of narcissists to be renowned and admired may occur through other means rather than through direct motivational pressure (Nurcahyono and Hanum, 2023; Chimonaki et al., 2023). This trend is consistent with the ToM approach, which suggests that social cognition allows narcissists to create self-serving, plausible narratives to justify unethical behavior without damaging their positive self-concept. The ability to justify unethical acts as acceptable or beneficial to themselves appears to be key to understanding how narcissistic characteristics are expressed in unethical intentions (Opriș et al., 2025; Doyle, 2020).

Machiavellian individuals, in turn, demonstrate a specific cognitive signature revealed by the mediation analysis, dominated by rationalization and perceived capability, and they appear to direct their strategic, manipulative tendencies toward processes of cognitive justification and beliefs about their ability to manipulate others (Elias, 2013; Harrison et al., 2018; Idensohn et al., 2025). Interestingly, opportunity perception and conscious intention formation were not notable pathways for Machiavellianism, which indicates that for these individuals the unethical pathway is less about actively identifying opportunities or forming explicit intentions and more about justifying manipulative actions as practical necessities and believing that they can perform them effectively (Smith et al., 2022; Almagtome and Abo-aljun, 2023). Such a tendency is consistent with the strategic and calculative nature of Machiavellianism; in such a worldview, manipulation becomes not only permissible and acceptable but also natural, and evolutionary theory frames Machiavellianism as an approach to social exploitation that manifests in modern contexts through these context-sensitive cognitive evaluations (Murthy and Gopalkrishnan, 2023; Latif et al., 2026; Volk et al., 2025).

The most significant conclusions are related to psychopathy, where simple depictions of this trait as merely impulsive and uncalculated, as the findings show, are insufficient, revealing instead a complex pattern of mediation through opportunity perception, rationalization, capability, and most compellingly, the formation of intentions (Bailey, 2017; Carré et al., 2020; Emerson and Smith, 2026). This implies that psychopaths develop distinctly immoral motives within the cognitive system of the Fraud Pentagon, involving cold cognitive evaluations of exploitable opportunities, developing rationalizations that reduce the value of others, evaluating their own abilities, and making premeditated plans to take action (Opriș et al., 2025; Pant, 2024). Importantly, similar to the other traits, motivation did not prove to be a significant pathway to psychopathy, which means that immoral conduct among psychopaths might not be caused by the perceived pressure or motivation but rather by the absence of emotional involvement in the decision-making process (Jones and Paulhus, 2011; Schilbach et al., 2020). These results indicate that even the cold and unemotional nature of psychopathy does not entirely undermine the familiar processes of cognitive justification, which implies that psychopathic people can use ToM abilities in instrumental ways that facilitate unethical actions (Opriș et al., 2025; Doyle, 2020).

Putting these findings together, a significant trend is apparent across all three Dark Triad traits: the consistent non-significance of motivation as a mediating factor suggests that, although motivation is conceptually a fundamental element of the Fraud Pentagon, in the case of accounting students with dark personality traits, unethical behavior may not be driven by perceived external or internal pressure but rather by cognitive evaluations of opportunity, rationalization, capability, and intention formation (Srirejeki et al., 2023). This subtlety extends theoretical understanding beyond simple trait–behavior relationships toward a more complex mechanism-driven explanation, namely, how ingrained personality systems manifest in specific context-dependent cognitive processes that eventually determine the unethical behavioral intentions of future accounting practitioners (Volk et al., 2025; Latif et al., 2026).

### Practical implications

This study has implications for regulators, policymakers, accreditation bodies, and agencies seeking to control fraud and unethical behavior. HEIs must recognize mechanisms for the prevention of fraud, and such mechanisms must highlight structural weaknesses and risk factors. Individuals with high scores on Dark Triad traits who occupy positions of power are more likely to commit fraud and engage in unethical behavior. Organizations must ensure fair screening processes for leaders and those occupying positions of power and assess the ethical climate and behavioral risks at regular intervals. Leadership development and training programs should include ethical orientation and personality assessment to monitor Dark Triad trait levels among individuals, as unchecked levels of Dark Triad personality traits may lead to unethical behavior. Auditors can benefit from the findings of this study by developing fraud risk evaluation frameworks that incorporate behavioral indicators such as manipulative leadership, financial red flags, excessive risk-taking, and arrogance. Fraud is multilayered and often reflects deviant personality traits (Thoma and Dong, 2014; Modic, et al 2018). Accordingly, the findings of this study may help organizations control misconduct, corruption, and fraudulent reporting that may emerge due to workers’ Dark Triad characteristics and may ultimately support smoother organizational operations. This study also has significant implications for prospective investors and stakeholders, both individual and institutional, to understand the factors that may lead to fraudulent actions in financial reporting. Our findings may also be useful for social and cultural development, from family environments to professional contexts. The findings further provide additional insight into understanding the impact of dark personality traits on behavioral accounting and fraud prevention. Organizations can also fund character-building workshops in public and private HEIs for accounting students, as today’s accounting students will become future professionals. Corrective actions should be taken if individuals with problematic dark personality traits are identified during the recruiting and selection phases. Similarly, organizations can advocate practical case studies illustrating how individuals with dark personality traits and strong fraudulent beliefs may impair organizational reputation. Finally, organizational policymakers, auditors, and regulators can foster an ethical culture, based on transparency, ethical leadership, and whistleblower protection to reduce unethical behavior.

### Theoretical implications

This study contributes to the literature on unethical behavior by integrating the Dark Triad and its attributes with the Fraud Pentagon and its five facets, which provides readers with a more in-depth explanation of unethical behavior. First, this study extends the Fraud Pentagon theory by suggesting that engagement in unethical practices may not be based solely on situational or organizational factors but may also be associated with personality traits. Second, this study also advances Dark Triad theory by contextualizing these personality traits within organizational and ethical decision-making frameworks. This study has novel implications and provides significant support to the accounting literature for examining and investigating unethical and fraudulent attitudes among accounting professionals, which may be useful for auditors, financial analysts, regulatory bodies, and corporate organizations, particularly in societies where corruption is prevalent. This is among the first studies in which accounting students responded to the SD3 scale and were simultaneously examined in relation to unethical behavior and Fraud Pentagon measures through the lens of evolutionary theory and theory of mind. Our research model and empirical findings provide significant contributions to educators, policymakers, and researchers by helping them understand why and how youngsters’ dark personality traits and the cognitive facets of the Fraud Pentagon influence unethical decision-making, and how such individuals may harm organizational performance, socio-political institutions, and social goodwill. Theory of Mind and Evolutionary Theory are employed in this study to strengthen the theoretical foundation for understanding the relationships between Dark Triad traits, unethical behavior, and the mediating role of the Fraud Pentagon. Understanding the cognitive and evolutionary factors that contribute to unethical behavior can help develop interventions, training programs, and policies that promote ethical decision-making and professional practices. Finally, this study empirically integrates Dark Triad theory and Fraud Pentagon theory with unethical behavior, which encourages future researchers to explore more psychologically informed theories of unethical behavior.

### Limitations and opportunities for further research

Obviously, this study has some limitations and presents opportunities for further research. Although comprehensive in scope, this study also encourages and facilitates further research. First and foremost, through its methodological approach and data collection techniques, this study presents opportunities for further research using prospective and more objective approaches to draw conclusions. The complexities associated with dark personality traits and diverse scenarios present scope for expanding sample diversity, as well as including additional factors, for example, ethical upbringing and social influences. The research also provides opportunities to further explore the mechanisms through which the Dark Triad influences ethical understanding in professional contexts. Second, as this was an initial effort to study the Dark Triad with the mediating influence of the Fraud Pentagon, future research could also incorporate the fourth dimension of the Dark Tetrad, “everyday sadism” (Paulhus et al., 2020). Third, this study was conducted using cross-sectional data; therefore, future studies could employ longitudinal designs to examine causal relationships over time. Similarly, this research framework can be extended to other populations, such as students, teachers, and professionals. Finally, cross-cultural and cross-industrial comparative studies are important to understand how varying ethical climates, regulatory environments, and professional socialization processes may moderate the mediated relationships identified in this study, thereby helping translate this psychological model into context-specific forensic and organizational tools.

## Conclusion

This study establishes that the Fraud Pentagon serves as a critical cognitive pathway linking Dark Triad personality traits to unethical intentions among accounting students. The findings confirm that narcissism, Machiavellianism, and psychopathy significantly predict unethical behavioral intentions through a mindset mediated by the cognitive appraisals of opportunity, rationalization, capability, and intention formation. Notably, the results clarify that even psychopathy’s influence is channeled through this calculated cognitive framework rather than mere impulsivity. The integration of evolutionary theory and theory of mind provides a robust explanation for these relationships, suggesting that dark personality traits, which may have evolved as socially exploitative strategies, find modern expression through the specific cognitive justifications captured by the Fraud Pentagon. This highlights how the capacity to understand others’ mental states can be co-opted to rationalize unethical conduct, thereby shifting the analysis from correlation to underlying mechanisms. These insights carry significant implications for accounting education and professional development, calling for ethics training that directly addresses the cognitive distortions and justifications facilitating unethical behavior, while underscoring the need for the profession to consider ethical predispositions and reasoning during recruitment.

## Data Availability

The raw data supporting the conclusions of this article will be made available by the authors, without undue reservation.
